# Supplementary material for: Detection of Polymorphisms in the MTNR1A Gene and Their Association with Reproductive Performance in Awassi Ewes
Source: Animals (Basel). 2021 Feb 23;11(2):583. doi: 10.3390/ani11020583 (PMC7926687; doi:10.3390/ani11020583)
Supplement: Supplementary file 1 [file animals-11-00583-s001.pdf]

Table S1. *MTNR1A* gene amplified regions and used primers

Amplified regions	Forward primers	Reverse primers
Promoter	GCACAAAAAGAAGCCAAGGA	TCAGGTGTCGCACTGTAACC
Promoter	TGTTCCTGGGAGAAGTCTGG	CATGCATCAAACCTGGACTG
Exon 1	CTCGACGCTCTGGGGAT	CCGAACAGGGAAGAGGTTG
Exon 2 and 3'UTR	GGCCCTAACCCATGTTTTCT	CTCCCACTCTGTTCCCTGAA

Table S2. PCR conditions for *MTNR1A* studied regions

	PCR steps	Promoter		Promoter		Exon 1		Exon 2 and 3' UTR	
	Initial denaturation	94°C	3min	94°C	3min	94°C	3min	94°C	3min
35 cycles	Denaturation	94°C	1min	94°C	1min	94°C	1min	94°C	1min
	Annealing	60°C	1min	60°C	1min	50°C	1min	54°C	1min
	Extension	72°C	1min	72°C	1min	72°C	30sec	72°C	1min
	Final elongation	72°C	10min	72°C	10min	72°C	10min	72°C	10min

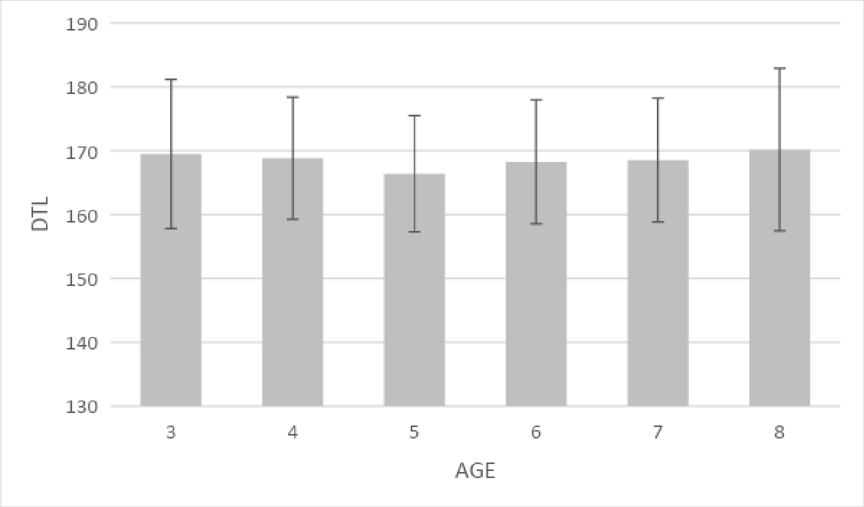

Figure S1. Graph showing the mean and the standard deviation of DTL divided by age, in the studied Lebanese Awassi population (n=165). DTL = Days To Lambing

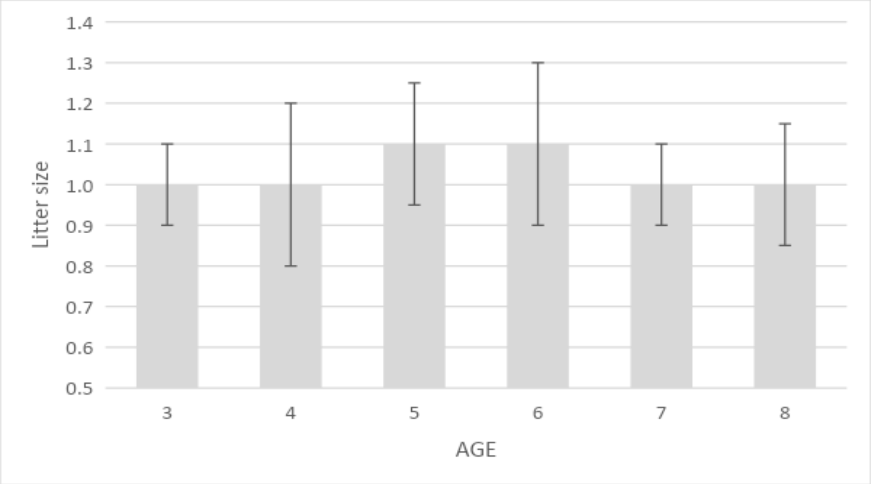

Figure S2. Graph showing the mean and the standard deviation of litter size divided by age, in the studied Lebanese Awassi population (n=165).

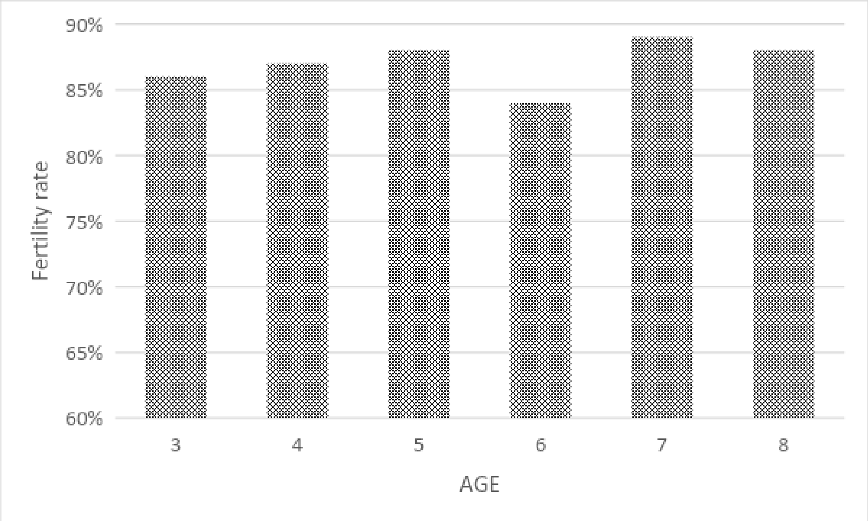

Figure S3. Graph showing fertility rate (%) divided by age, in the studied Lebanese Awassi population (n=165).
